# The association between oral dryness and use of dry-mouth interventions in Sjögren’s syndrome patients

**DOI:** 10.1007/s00784-021-04120-2

**Published:** 2021-08-10

**Authors:** Z. Assy, F. J. Bikker, O. Picauly, H. S. Brand

**Affiliations:** Department of Oral Biochemistry, Academic Centre for Dentistry Amsterdam, University of Amsterdam and VU University Amsterdam, Gustav Mahlerlaan, 3004, Amsterdam, 1081 LA the Netherlands

**Keywords:** Sjögren’s syndrome, Dry mouth, Xerostomia, Xerostomia inventory, Bother Index, Dry-mouth interventions

## Abstract

**Objective:**

Sjögren’s syndrome patients use different dry-mouth interventions for the relieve of their oral dryness. Recently, it was shown that patients with dry-mouth complaints have regional differences in perceived intra-oral dryness. Therefore, the aim of the present study was to investigate whether the use of dry-mouth interventions is related to the perceived regional oral dryness.

**Materials and methods:**

A cross-sectional study was performed among Sjögren’s patients. Volunteers could anonymously administer various questionnaires, including the Regional Oral Dryness Inventory (RODI), Xerostomia Inventory (XI), Bother Index (BI) and a list of dry-mouth interventions.

**Results:**

Sjögren’s syndrome patients use a wide variety for the relieve of oral dryness. “Drinking water’’ and “moistening the lips’’ were used most frequently. Dry-mouth interventions, “drinking water’’, “rinsing of the mouth”, and “drinking small volumes” had significant associations with the RODI-scores of the posterior palate, and anterior and posterior tongue, respectively. On the other hand, “using mouth gel’’ had a significant association with the RODI-scores of the inside cheeks.

**Conclusion:**

Sjögren’s syndrome patients are more likely to use mouth gels when their inside cheeks were experienced as most dry, while they drank water, rinsed their mouth or drank small volumes if the posterior palate, and anterior and posterior tongue were considered as dry. It can be concluded that intra-oral dryness affects dry-mouth perception and thereby also the use of the various dry-mouth interventions.

**Clinical relevance:**

The therapeutic choice of dry-mouth interventions by Sjögren’s syndrome patients seems to some extent to be related to dryness at specific intra-oral regions.

## Introduction

Sjögren’s syndrome is an autoimmune disease that affects the exocrine lacrimal and salivary glands [1, 2]. As a result of progressive immune-mediated damage to the salivary glands, Sjögren’s syndrome is associated with hyposalivation and xerostomia [1]. Both hyposalivation and xerostomia may induce comorbidities such as difficulty with swallowing, speaking and sleeping. Loss of the protective and antimicrobial properties of saliva may also increase the risk of oral diseases such as dental caries and oral candidiasis [1, 3]. This negatively affects the oral health and the quality of life [1, 4]. In order to relieve their dry mouth complaints, Sjögren’s syndrome patients seek for effective care and treatment.

In early stages of Sjögren’s syndrome, when residual salivary function is still present, salivary flow can be stimulated, e.g. by the use of lozenges and chewing gums. Upon prescription, systemic pharmacotherapies, such as pilocarpine or cevimeline, might be used [4–6]. Alternatively, electrostimulation of the salivary glands and acupuncture have been reported to increase saliva production [4, 5]. However, when the salivary function is irreversibly impaired, only the use of saliva substitutes remains for the relieve of oral problems. For this purpose, a wide range of salivary substitutes such as mouth sprays, gels and mouthwashes is available.

Despite the fact that several dry-mouth interventions are available, their effectiveness seems to be limited. Although the use of pilocarpine is associated with a reduction in dry mouth symptoms, the effect size, clinical significance and duration of the effect remain unclear [4]. Furthermore, for cevimeline and electrostimulation, there is limited evidence with respect to increasing the salivary flow in Sjögren’s syndrome patients [4]. Besides, adverse events such as nausea, sweating or headache are commonly reported for individuals taking pilocarpine and cevimeline [4]. Additionally, these pharmacotherapies may be contraindicated in patients with comorbidity like chronic respiratory, cardiovascular or renal disease [6]. Taken together, there is no robust evidence that any of the treatments known is fully effective or leads to a widely supported satisfaction to relieve dry mouth complains [5–7]. As a consequence, therapeutic advice of healthcare professionals to patients with Sjögren’s syndrome is difficult and generally based on a combination of dentist’s opinion, scientific literature, patients’ personal experience and availability of products [4]. The advice is usually related to the overall oral dryness severity. However, we have recently shown that there are significant regional differences in perceived intra-oral dryness [8, 9]. Dry-mouth patients experienced the oral dryness of the posterior palate as most severe, while the floor of the mouth and the inside cheeks were experienced less dry. Therefore, the aim of the present study was to investigate possible associations between the use of dry-mouth interventions and the perceived oral dryness, both overall and regional, of Sjögren’s syndrome patients. We anticipate that this information will contribute in developing more tailored advice about dry-mouth intervention(s) for Sjögren’s syndrome patients.

## Materials and method

### Study design

A cross-sectional study was performed among Sjögren’s syndrome patients who visited the annual meeting of the Dutch Sjögren Patients Federation on October 5th, 2019 (Dutch: Nederlandse Vereniging van Sjögren Patiënten). Volunteers could anonymously fill in the questionnaire described below, and return it in a designated mailbox during the meeting or return then questionnaire by mail using an enclosed prepaid envelope.

The local Ethics Review Committee of the Academic Centre for Dentistry Amsterdam (ACTA) confirmed that the Medical Research Involving Human Subjects Act (WMO) did not apply to this study (protocol number 201930). The reporting of this study conforms to the STrengthening the Reporting of OBservational studies in Epidemiology (STROBE) statement [10].

### Study variables

The questionnaire, developed for this study, consists of five parts. First, some general questions with regard to age, sex and year in which Sjögren’s syndrome had been diagnosed by a physician.

Secondly, the Regional Oral Dryness Inventory (RODI) questionnaire was used to determine differences in dry-mouth perception at different intra-oral locations. The RODI questionnaire contains nine schematic illustrations of different locations in the oral cavity [8, 9]. Four illustrations represent areas in the upper jaw: the upper lip, the posterior part of the palate (from the rugae up to the end of the soft palate), the anterior part of the palate (including the rugae) and the inside part of the cheeks. Four other illustrations represent areas in the lower jaw: the lower lip, the anterior part of the tongue (from the tip of the tongue up to the vallate papilla), the posterior part of the tongue (from the vallate papilla up to end of the tongue) and the floor of the mouth. Finally, one illustration represents the pharynx. At each location, the patient can indicate the severity of the intra-oral dryness on a 5-point Likert scale ranging from 1—"No dryness" to 5—"Severe dryness” [8, 9].

The third part was the Xerostomia Inventory (XI), consisting of 11 items on a 5-point Likert scale ranging from 1—"Never" to 5—"Very often”. The items concern patients’ oral dryness and mouthfeel. Per item, patients indicate how often they experience problems regarding mouthfeel and oral dryness. The scores of the 11 items are summed to produce a total XI-score that ranges between 11 (no xerostomia) and 55 (extreme xerostomia) [11].

The fourth part consisted of the Bother Index (BI). In the BI, the patient is asked to rate the severity of dry mouth on a scale from 0 to 10 [12–16].

Finally, the questionnaire included a list of potential interventions to relieve the feeling of a dry mouth [17]. These interventions are summed up in Table 2 and divided into two categories: the frequently (> 20%) and less frequently used (< 20%) interventions. The participants could indicate with yes/no which options they apply for the relieve of their dry mouth. With the option “using other interventions’’, they could report additional interventions applied not listed in the questionnaire. Because some respondents did not answer all items of the questionnaires, the total number for items may differ.

### Data analysis

The data were statistically analyzed with SPSS, version 26.0 (IBM Corp SPSS statistics, Armonk, NY, USA). The Shapiro–Wilk test was used to assess the normality of the data. As not all variables were normally distributed, the data are presented as medians and their interquartile range (IQR). To clarify relatively small differences, the mean and standard deviation (SD) are also reported.

A Friedman test was conducted for the RODI-scores of the total study population, followed by a Wilcoxon signed-rank test as a post hoc procedure.

The possible relationships between frequently used dry-mouth interventions and the perceived oral dryness (RODI and XI) and patients’ discomfort (BI) were investigated initially by using a univariate analysis, using Mann–Whitney *U* tests. Only the significant interventions found in the univariate analysis were further explored in the multivariate analysis, the binary logistic regression. The dry-mouth interventions were considered as dependent variable and the total XI-score, BI-score and RODI-scores of the nine intra-oral regions were considered as independent variables. To identify the degree of multicollinearity among the independent variables, the variance inflation factor (VIF) was calculated. The VIF for these variables was < 5, which indicates that there is no multicollinearity present among these variables [18, 19], so they do not influence each other.

The backward conditional method was used to analyze these independent variables. If there was a significant association between a dry-mouth intervention and one or more independent variables, then the odds ratio and the 95% confidence interval (95% CI) were reported. Furthermore, the last step of the Omnibus test chi-square, and the Hosmer and Lemeshow (H–L) test chi-square including their degree of freedom (df) and their *p*-values were reported. Also, the Coxx & Snell R square and the Nagelkerke R square were mentioned, if the association was significant.

All significance levels (*α*) were set at 0.05.

## Results

At the yearly meeting of the patient federation, 176 questionnaires were distributed. In total, 91 questionnaires were returned, which results in a response rate of 51.7%. Most of the respondents were female (*N* = 81, 89.0%), while 6 of respondents were male and 4 did not indicate their gender. The mean age of the respondents was 64 ± 10 years, ranging from 35 to 84 years. Almost all patients (*N* = 87, 95.6%) reported that they had been diagnosed with Sjögren’s syndrome by a physician, while 4 respondents did not answer this question. After excluding these four respondents, the final study population consisted of 78 females, 6 males and 3 without any indication of their gender. Removal of these respondents did not affect the mean age.

### Perceived oral dryness and patients’ oral discomfort

The perceived oral dryness at various intra-oral locations, as determined with the RODI questionnaire, is presented in Table 1. Perceived oral dryness in the total study population differed significantly among the nine intra-oral regions (Friedman test *p* < 0.05, followed by Wilcoxon signed-rank test). It was found that the perceived intra-oral dryness was found to be most severe for the posterior palate and the pharynx. In contrast, the floor of the mouth and the inside of the cheeks were experienced as least dry.

**Table 1:** Perceived oral dryness in nine intra-oral regions as determined with the Regional Oral Dryness Inventory (RODI) in the study population. Data are presented as median with corresponding interquartile range (IQR) and as a mean with standard deviation (SD). *N* indicates the total number of respondents for each intra-oral region

Intra-oral regions	Mean	SD	Median	IQR	*N*
Upper lip	3.25	1.00	3.0	3.0–4.0	85
Inside cheeks	3.13	1.04	3.0	2.0–4.0	86
Anterior palate^b^	3.40	1.04	3.0	3.0–4.0	86
Posterior palate^a,b,c^	3.67	0.99	4.0	3.0–4.0	86
Lower lip^c,d^	3.19	0.98	3.0	3.0–4.0	83
Floor of the mouth^a,c,d^	3.02	1.09	3.0	2.0–4.0	83
Anterior tongue^a,e,f^	3.46	1.10	4.0	3.0–4.0	83
Posterior tongue^a,b,e,f^	3.54	1.00	4.0	3.0–4.0	84
Pharynx^a,b,e,f^	3.61	1.03	4.0	3.0–4.0	83

^a^Wilcoxon signed-rank tests: *p* < 0.05 vs. upper lip

^b^Wilcoxon signed-rank tests: *p* < 0.05 vs. inside cheeks

^c^Wilcoxon signed-rank tests: *p* < 0.05 vs. anterior palate

^d^Wilcoxon signed-rank tests: *p* < 0.05 vs. posterior palate

^e^Wilcoxon signed-rank tests: *p* < 0.05 vs. lower lip

^f^Wilcoxon signed-rank tests: *p* < 0.05 vs. floor of the mouth

^g^Wilcoxon signed-rank tests: *p* < 0.05 vs. anterior part of the tongue

^h^Wilcoxon signed-rank tests: *p* < 0.05 vs. posterior part of the tongue

The overall dry-mouth feeling was quantified with the XI. The mean total XI-score of the total study population was 42.8 ± 8.7 with a median score of 45.0 and IQR of 38.0–48.5 (*N* = 85). Patients dry-mouth discomfort as measured with BI had a mean of 7.1 ± 2.4 with a median score of 8.0 and IQR of 6.0–9.0 (*N* = 87).

### Dry-mouth interventions strategies

Most respondents use one or more interventions for the relieve of their dry mouth complaints (see Table 2). The most frequently used interventions (> 20%) to relieve dry mouth complaints were “drinking water’’ and “moistening the lips’’. Less frequently used interventions (< 20%) by Sjögren’s syndrome patients were “keeping lemon slices in the mouth’’ and “putting olive oil in the mouth’’. Most reported “using other medications’’ that Sjögren’s syndrome patients used included the use of Xylimelts®, oral adhering discs that release xylitol and cellulose gum upon use. The spontaneously reported “using other interventions’’ included “drinking chocolate milk”,”using mouth wash”, “using specialized toothpaste” and “using different kind of candies”.

**Table 2:** Frequently (> 20%) and less frequently used (< 20%) interventions by Sjögren’s syndrome patients to relieve dry-mouth symptoms. Data are expressed as percentages

Frequently used intervention for dry mouth	%	Less frequently used intervention for dry mouth	%
**Drinking water**	90.5	**Focusing on other activities**	13.1
**Moistening the lips**	72.6	**Using other interventions**	12.2
**Drinking tea**	60.7	**Using other medications**	11.9
**Rinsing of the mouth**	50.0	**Using pilocarpine**	8.3
**Chewing gum**	48.8	**Drinking lemonade**	4.8
**Drinking small volumes**	48.8	**Drinking soft drinks**	3.6
**Using mouth gel**	42.9	**Using acupuncture**	3.6
**Eating fruit**	40.5	**Drinking beer**	3.6
**Using mouth spray**	27.4	**No intervention**	3.6
**Drinking coffee**	25.0	**Sucking ice cubes**	2.4
**Sucking sour candies**	23.8	**Putting olive oil in the mouth**	1.2
		**Keeping lemon slices in the mouth**	1.2

### Univariate analysis of the association of oral dryness and patients’ discomfort with dry-mouth interventions strategies

The association between the perceived oral dryness of Sjögren’s syndrome patients and the frequently used intervention strategies (used by more than 20% of the study population) to relieve dry mouth was further explored. In Tables 3 and 4, the associations are presented between the use of these interventions and the perceived dryness at different intra-oral locations (RODI-scores). Respondents who rinsed their mouth and those who refrained from rinsing their mouth showed significant differences in RODI-scores for all intra-oral regions except for the anterior palate. The RODI-scores of patients who use water were higher for all intra-oral regions except for the anterior palate, lower lip and the anterior tongue than the RODI-scores of patients who did not drink water. For other dry-mouth interventions, only a few regions showed significant differences between patients who applied an intervention and patients who did not apply that intervention. Interestingly, for “using mouth gel’’, significant differences were only observed for the inside cheeks and the anterior palate. Intra-oral dryness was not related to the use of the following dry-mouth interventions: “sucking sour candies’’, “chewing gum’’, “eating fruit’’, “moistening the lips’’ and “using mouth spray’’.

**Table 3:** The RODI-scores of the upper jaw for Sjögren’s syndrome patients who report the use of a specific intervention for the relieve of dry mouth complaints versus patients who do not use that intervention. Data are expressed as mean scores with standard deviation (SD)

Intervention	Use	Upper lip	Inside cheek	Anterior palate	Posterior palate
Drinking water	Yes No	3.4 ± 0.9* 2.3 ± 1.1	3.2 ± 1.0* 2.1 ± 1.1	3.5 ± 1.0 2.4 ± 1.5	3.8 ± 0.9** 2.4 ± 1.4
Moistening the lips	Yes No	3.4 ± 1.0 3.0 ± 1.1	3.2 ± 1.0 3.0 ± 1.1	3.4 ± 1.1 3.4 ± 1.1	3.7 ± 0.9 3.6 ± 1.1
Drinking tea	Yes No	3.2 ± 0.9 3.3 ± 1.1	3.0 ± 1.0 3.3 ± 1.1	3.3 ± 1.0* 3.6 ± 1.1	3.6 ± 0.9 3.7 ± 1.1
Rinsing of the mouth	Yes No	3.6 ± 0.9** 3.0 ± 1.0	3.5 ± 1.0** 2.8 ± 1.0	3.6 ± 1.0 3.2 ± 1.1	3.9 ± 0.9** 3.4 ± 1.0
Chewing gum	Yes No	3.4 ± 1.0 3.2 ± 1.0	3.2 ± 1.1 3.1 ± 1.0	3.6 ± 1.0 3.2 ± 1.1	3.6 ± 0.9 3.7 ± 1.0
Drinking small volumes	Yes No	3.4 ± 0.9 3.2 ± 1.1	3.3 ± 1.0 2.9 ± 1.1	3.5 ± 0.9 3.3 ± 1.28	3.9 ± 0.8 3.4 ± 1.1
Using mouth gel	Yes No	3.5 ± 0.8 3.1 ± 1.1	3.5 ± 0.8** 2.9 ± 1.1	3.7 ± 0.9* 3.2 ± 1.1	3.8 ± 1.0 3.5 ± 1.0
Eating fruit	Yes No	3.4 ± 0.9 3.2 ± 1.1	3.2 ± 1.0 3.1 ± 1.1	3.7 ± 1.0 3.2 ± 1.1	3.9 ± 0.8 3.5 ± 1.1
Using mouth spray	Yes No	3.3 ± 0.9 3.3 ± 1.0	3.3 ± 1.1 3.1 ± 1.1	3.5 ± 1.0 3.4 ± 1.1	3.7 ± 1.0 3.7 ± 1.0
Drinking coffee	Yes No	3.4 ± 0.9 3.2 ± 1.0	3.1 ± 0.8 3.1 ± 1.1	3.6 ± 0.8 3.3 ± 1.1	3.9 ± 0.8 3.6 ± 1.0
Sucking sour candies	Yes No	3.2 ± 0.9 3.3 ± 1.0	3.1 ± 0.8 3.1 ± 1.1	3.6 ± 1.1 3.3 ± 1.0	3.7 ± 0.9 3.6 ± 1.0

^*^p < 0.05 compared to the RODI-score of patients who do not use the intervention, Mann–Whitney *U* test

^**^p < 0.01 compared to the RODI-score of patients who do not use the intervention, Mann–Whitney *U* test

**Table 4:** The RODI-scores of the lower jaw and pharynx for Sjögren’s syndrome patients who report the use of a specific intervention for the relieve of dry-mouth complaints versus patients who do not use that intervention. Data are expressed as mean scores with standard deviation (SD)

**Intervention**	**Use**	**Lower lip**	**Floor of the mouth**	**Anterior tongue**	**Posterior tongue**	**Pharynx**
Drinking water	Yes No	3.3 ± 0.9 2.6 ± 1.3	3.1 ± 1.1 2.0 ± 1.2*	3.5 ± 1.1 2.6 ± 1.3	3.6 ± 0.9** 2.4 ± 1.1	3.7 ± 0.9* 2.4 ± 1.5
Moistening the lips	Yes No	3.3 ± 0.9 2.9 ± 1.1	3.1 ± 1.1 2.8 ± 1.2	3.5 ± 1.1 3.3 ± 1.2	3.5 ± 1.0 3.6 ± 1.1	3.7 ± 1.0 3.4 ± 1.1
Drinking tea	Yes No	3.2 ± 1.0 3.2 ± 1.0	3.0 ± 1.1 3.1 ± 1.2	3.4 ± 1.1 3.5 ± 1.2	3.6 ± 0.9 3.4 ± 1.1	3.7 ± 1.0 3.6 ± 1.1
Rinsing of the mouth	Yes No	3.5 ± 0.9* 3.0 ± 1.1	3.4 ± 0.9** 2.7 ± 1.2	3.9 ± 0.8** 3.0 ± 1.2	3.8 ± 0.9* 3.3 ± 1.1	3.9 ± 1.0* 3.4 ± 1.1
Chewing gum	Yes No	3.3 ± 1.1 3.1 ± 0.9	3.2 ± 1.2 2.9 ± 1.0	3.6 ± 1.1 3.3 ± 1.1	3.6 ± 1.0 3.5 ± 1.0	3.5 ± 1.0 3.8 ± 1.1
Drinking small volumes	Yes No	3.3 ± 1.0 3.1 ± 1.0	3.2 ± 1.0 2.8 ± 1.2	3.7 ± 1.0 3.2 ± 1.2	3.8 ± 0.9** 3.2 ± 1.0	3.9 ± 0.8* 3.3 ± 1.2
Using mouth gel	Yes No	3.5 ± 0.9 3.0 ± 1.0	3.3 ± 1.0 2.8 ± 1.2	3.8 ± 1.0 3.2 ± 1.2	3.8 ± 1.1 3.4 ± 0.9	3.7 ± 1.1 3.5 ± 1.0
Eating fruit	Yes No	3.3 ± 0.9 3.2 ± 1.0	3.2 ± 1.0 2.9 ± 1.2	3.8 ± 0.8 3.2 ± 1.3	3.7 ± 0.9 3.4 ± 1.1	3.8 ± 1.0 3.5 ± 1.1
Using mouth spray	Yes No	3.5 ± 0.8 3.1 ± 1.0	3.2 ± 1.1 2.9 ± 1.1	3.7 ± 0.9 3.4 ± 1.2	3.7 ± 1.0 3.5 ± 1.0	3.9 ± 1.1 3.5 ± 1.0
Drinking coffee	Yes No	3.5 ± 0.9 3.1 ± 1.0	3.5 ± 0.7* 2.9 ± 1.2	3.6 ± 1.0 3.4 ± 1.1	3.9 ± 0.8 3.4 ± 1.0	3.9 ± 1.0 3.5 ± 1.1
Sucking sour candies	Yes No	3.2 ± 0.8 3.2 ± 1.0	3.1 ± 0.7 3.0 ± 1.2	3.5 ± 0.8 3.4 ± 1.2	3.5 ± 0.8 3.5 ± 1.1	3.8 ± 0.6 3.6 ± 1.1

^*^p < 0.05 compared to the RODI-score of patients who do not use the intervention, Mann–Whitney *U* test

^**^p < 0.01 compared to the RODI-score of patients who do not use the intervention, Mann–Whitney *U* test

Table 5 shows the association between the total XI-scores and frequently used dry-mouth intervention strategies. Interventions that are associated with significant higher total XI-scores are “rinsing of the mouth”, “drinking water”, “eating fruit” and “using mouth gel”, indicating that the patients who use these interventions suffer from more severe overall dry mouth than patients who refrain from these interventions. All other interventions did not show any relation with the XI-scores.

**Table 5:** The total XI-scores of Sjögren’s syndrome patients who report the use of a specific intervention for the relieve of dry mouth complaints versus patients who do not use that intervention. Data are expressed as mean scores with standard deviation (SD)

Intervention	XI-total of patients who use intervention (mean ± SD)	XI-total of patients who do not use intervention (mean ± SD)
Drinking water	43.7 ± 7.5*	32.9 ± 13.4
Moistening the lips	43.5 ± 8.2	40.5 ± 10.1
Drinking tea	42.4 ± 7.7	43.1 ± 10.3
Rinsing of the mouth	45.9 ± 6.6**	39.5 ± 9.6
Chewing gum	43.7 ± 7.6	41.7 ± 9.7
Drinking small volumes	44.4 ± 6.2	40.9 ± 10.6
Using mouth gel	45.4 ± 6.1*	40.5 ± 9.9
Eating fruit	45.8 ± 6.7**	40.6 ± 9.4
Using mouth spray	44.6 ± 7.3	41.9 ± 9.2
Drinking coffee	44.8 ± 7.3	42.0 ± 9.2
Sucking sour candies	45.7 ± 4.8	41.8 ± 9.5

^*^p < 0.05 compared to XI-total of patients who do not use the intervention, Mann–Whitney *U* test

^**^p < 0.01 compared to XI-total of patients who do not use the intervention, Mann–Whitney *U* test

Table 6 presents the BI-scores of patients who apply frequently used dry-mouth interventions versus patients who do not use these interventions. Only Sjögren’s patients who rinsed their mouth and/or who drank water had significantly higher BI-scores and thereby more dry-mouth discomfort than patients who refrained from these interventions. For all other interventions, there were no significant differences between patients who use a specific intervention or those who refrain from that intervention.

**Table 6:** The BI-scores of Sjögren’s syndrome patients who report the use of a specific intervention for the relieve of dry mouth complaints versus patients who do not use that intervention. Data are expressed as mean scores with standard deviation (SD)

Intervention	BI-score of patients who use intervention (mean ± SD)	BI-score of patients who do not use intervention (mean ± SD)
Drinking water	7.4 ± 2.1**	3.9 ± 3.5
Moistening the lips	7.1 ± 2.5	7.0 ± 2.4
Drinking tea	6.8 ± 2.3	7.5 ± 2.7
Rinsing of the mouth	8.0 ± 1.8**	6.1 ± 2.7
Chewing gum	7.1 ± 2.5	7.0 ± 2.5
Drinking small volumes	7.7 ± 1.8	6.4 ± 2.8
Using mouth gel	7.6 ± 2.1	6.6 ± 2.6
Eating fruit	7.6 ± 1.9	6.7 ± 2.7
Using mouth spray	7.5 ± 1.5	6.9 ± 2.7
Drinking coffee	7.2 ± 1.8	7.0 ± 2.7
Sucking sour candies	7.7 ± 2.3	6.9 ± 2.5

^*^p < 0.05 compared to BI-score of patient who do not use the intervention, Mann–Whitney *U* test

^**^p < 0.01 compared to BI-score of patients who do not use the intervention, Mann–Whitney *U* test

### Multivariate analysis of the association of oral dryness and patients’ discomfort with dry-mouth interventions strategies

In Table 7, the odds ratios for the dry-mouth interventions are reported. Interestingly, general interventions such as “drinking water’’, “rinsing the mouth’’ and “drinking small volumes’’ had significant odds ratios for respectively the RODI-scores of the posterior palate, anterior and posterior tongue areas. This result indicates that patients having more severe dryness at these intra-oral regions would more likely use of these general dry-mouth interventions. For “using a mouth gel’’, there was only a significant association with the RODI-scores of the inside cheeks. As for “eating fruit’’, there was an association with the total XI-score, indicating that overall oral dryness could influence the use of the dry-mouth intervention “eating fruit’’. Only “drinking coffee’’ had significant associations with two intra-oral regions, the inside cheeks and the floor of the mouth. However, the RODI-score of the inside cheeks was below 1 (0.25), while the RODI-score of the floor of the mouth was larger than 1 (2.82). This indicates that higher RODI-scores for the floor of the mouth and lower scores of the inside cheeks will probably affect drinking coffee by Sjögren’s patients. The dry-mouth intervention “drinking tea’’ did not have any significant association with any of the included independent variables. Also, the independent variable, BI-score, did not have any significant association with any dry-mouth intervention.

**Table 7:** The odds ratio of several independent variables (RODI-scores, total XI-scores, BI-score) for the significant interventions after univariate analysis. The odds ratio including the 95% CI is reported. For the significant associations, also the last step of the Omnibus and H–L test chi-square including their df and *p*-values were reported. Furthermore, the Coxx & Snell and the Nagelkerke R square were mentioned

	**Drinking water**	**Drinking tea**	**Rinsing of the mouth**	**Drinking small volumes**	**Using mouth gel**	**Eating fruit**	**Drinking coffee**
RODI-score of upper lip	NS	NS	NS		NS	NS	NS
RODI-score of inside cheeks	NS	NS	NS	NS	1.97 (1.19–3.27)^**,d^	NS	0.25 (0.09–0.70)^**,f^
RODI-score of anterior palate	NS	NS	NS	NS	NS	NS	NS
RODI-score of posterior palate	4.90 (1.70–14.08)^**,a^	NS	NS	NS	NS	NS	NS
RODI-score of lower lip	NS	NS	NS	NS	NS	NS	NS
RODI-score of floor of the mouth	NS	NS	NS	NS	NS	NS	2.82 (1.10–7.19)^*,f^
RODI-score of anterior tongue	NS	NS	1.90 (1.09–3.30)^*,b^	NS	NS	NS	NS
RODI-score of posterior tongue	NS	NS	NS	2.00 (1.21–3.31)^**,c^	NS	NS	NS
RODI-score of pharynx	NS	NS	NS	NS	NS	NS	NS
Total XI-score	NS	NS	NS	NS	NS	1.09 (1.02–1.17)^**,e^	NS
BI-score	NS	NS	NS	NS	NS	NS	NS

*NS* none of the independent variables was significant

Binary Logistic regression: *p < 0.05

Binary Logistic regression: **p < 0.01

^a^H-L test *χ*^2^ = 12.4, df = 2, *p* < 0.01; Omnibus test *χ*^2^ = 12.5, df = 1, *p* < 0.01; Cox & Snell *R*^2^ = 0.15; Nagelkerke *R*^2^ = 0.33

^b^H-L test *χ*^2^ = 11.6, df = 6, *p* > 0.05; Omnibus test *χ*^2^ = 16.3, df = 2, *p* < 0.01; Cox & Snell *R*^2^ = 0.19; Nagelkerke *R*^2^ = 0.26

^c^H-L test *χ*^2^ = 2.5, df = 2, *p* > 0.05; Omnibus test *χ*^2^ = 8.3, df = 1, *p* < 0.01; Cox & Snell *R*^2^ = 0.10; Nagelkerke *R*^2^ = 0.14

^d^H-L test *χ*^2^ = 3.7, df = 3, *p* > 0.05; Omnibus test *χ*^2^ = 7.9, df = 1, *p* < 0.01; Cox & Snell *R*^2^ = 0.10; Nagelkerke *R*^2^ = 0.13

^e^H-L test *χ*^2^ = 8.2, df = 7, *p* > 0.05; Omnibus test *χ*^2^ = 8.6, df = 1, *p* < 0.01; Cox & Snell *R*^2^ = 0.11; Nagelkerke *R*^2^ = 0.14

^f^ H–L test χ^2^ = 4.2, df = 8, p > 0.05; Omnibus test χ^2^ = 14.3, df = 3, p < 0.01; Cox & Snell R^2^ = 0.17; Nagelkerke R^2^ = 0.25

## Discussion

The present study was designed to explore the possible associations between the perceived (regional) oral dryness of Sjögren’s syndrome patients, and patients’ use of dry-mouth interventions. Sjögren’s syndrome patients use various interventions to relieve their oral dryness. Of those interventions, “drinking water” and “moistening the lips’’ were the most frequently used. Besides, there were some clear associations between perceived oral dryness and some interventions applied, illustrated by the significant odds ratios between general dry-mouth interventions, “drinking water’’, “rinsing of the mouth”, and “drinking small volumes” with the RODI-scores of the posterior palate, anterior and posterior tongue, respectively. On the other hand, “using mouth gel’’ was significantly associated with the RODI-scores of the inside cheeks. This observation could indicate that the use of these dry-mouth interventions is affected by the intra-oral dryness, measured by the RODI questionnaire.

The Sjögren’s syndrome patients in the current study experienced the posterior palate and the pharynx as most dry. This observation could be explained by the fact that several factors make the hard palate more susceptible to oral dryness compared with other intra-oral locations. These factors include paucity of palatal glands, gravity, and evaporation during open-mouth breathing [20–22]. Besides, it is envisaged that saliva-related changes also contribute to the dry mouth feeling of Sjögren’s syndrome patients: an altered sialochemical composition, such as higher concentrations of sodium, chloride and phosphate [23]; a higher protein concentration on the palate [24]; a significantly reduced saliva film on the hard palate; a reduced spinnbarkheit of unstimulated whole saliva; and an altered glycosylation of salivary mucins [16]. All these factors seem to negatively influence the wetting of the posterior palate and the pharynx.

In contrast, the Sjögren’s syndrome patients experienced the floor of the mouth and inside cheeks as least dry. These regions include the orifices of the major salivary gland [20]. Because of their proximity to the orifices of the salivary glands, the saliva film in these regions is probably more moisturizing than the saliva film on the palate [21, 25–27].

The results in the current study are consistent with our previous study which reported the perceived intra-oral dryness for various dry-mouth patients [8], including Sjögren’s patients as well as patients with polypharmacy and patients treated with radiotherapy. In that previous study, it was also found that the posterior palate was also the most dry in Sjögren’s syndrome patients, while the floor of the mouth and the inside cheeks were experienced as least dry [8]. This supports the suggestion that use of the RODI might add in screening or diagnosis of Sjögren syndrome.

The current study found that the use of dry-mouth interventions is influenced by intra-oral dryness (RODI questionnaire) of Sjögren’s patients. For almost all dry-mouth interventions, there was a significant association with the RODI-scores except for “eating fruit’’ (Table 7). Only “eating fruit’’ was significantly associated with the overall mouth dryness (total XI-score); however, the odds ratio was only slightly above 1 (1.09). While for all other associations between dry-mouth interventions and RODI-scores, the odds ratios were around 2 or above (Table 7). On the other hand, patients’ discomfort was not significantly associated with any dry-mouth interventions. These results show that the intra-oral dryness, measured by the RODI questionnaire, can be a helpful tool in advising dry-mouth interventions for Sjögren’s syndrome patients.

An interesting significant association could be seen for the dry-mouth interventions “drinking water’’, “rinsing of the mouth”, and “drinking small volumes’’ with some intra-oral regions. However, it is expected that these generic dry interventions would be significantly associated with the overall mouth dryness (XI-score) and not with the intra-oral dryness. In a previous study, it was found that the XI-scores of Sjögren’s patients had the highest correlations with the RODI-scores of the posterior palate, anterior and posterior tongue, and floor of the mouth [8]. When looking to the other dry-mouth patients, it was found that the RODI-scores of the anterior and posterior tongue and the floor of the mouth had the highest correlations with total XI-scores [8]. This finding indicates that the tongue and possibly also the posterior palate play an important role in dry-mouth perception. A different study that used the Clinical Oral Dryness Score (CODS), a clinical tool to semi-quantitatively assess oral dryness, found that the items “fissured or depapillated tongue’’ and “lack of saliva pooling in the floor of the mouth’’ are signs of hyposalivation [28]. Other clinical features of their study, such as a mirror sticking to the tongue, a lack of saliva pooling in the floor of the mouth and a tongue showing loss of papillae, can be associated with a moderate but significant reduction in mucosal wetness [28]. Taken together, this suggests that the tongue might play an important role in dry-mouth perception. This may explain why Sjögren’s patients have a significant association between “rinsing of the mouth”, “drinking small volumes” and the RODI-scores of the anterior and posterior tongue, respectively. The significant association between “drinking water’’ and the RODI-scores of the posterior palate is explained by the high RODI-scores of this region. Of all intra-oral regions, the posterior palate was considered the most dry compared to all other intra-oral regions except the anterior and posterior tongue and the pharynx (see Table 1). This result shows that dryness of the posterior palate in combination with dryness of the anterior and posterior tongue seems to play a major role in choosing a dry-mouth intervention, much more than the total XI-score.

Other interesting findings were the significant associations between “using mouth gel’’ and the RODI-score of the inside cheeks (see Table 7). As seen in Table 1, the inside cheeks were considered as least dry region. However, when this region becomes more dry (Table 3, RODI-score ≥ 3.5), patients tend to use a mouth gel that can be applied to this region to relieve its dryness.

The frequently used dry-mouth interventions by Sjögren’s syndrome patients were “drinking water’’ and “moistening the lips’’. Drinking water was the most used (90.5%) intervention compared to all other dry-mouth interventions. As mentioned earlier by several systematic reviews, dry mouth products are not effective to relieve dry mouth [4, 6, 7]. Especially salivary substitutes, such as mouth gels and sprays, are not effective in reducing dry mouth symptoms or increasing the salivary flow [4, 6, 7]. This is in line with previous research that interviewed Sjögren’s syndrome patients in the Netherlands about their saliva substitutes usage [29]. These patients reported that they discontinued use of saliva substitutes after a short period of time due to lack of effectiveness [29]. Possibly for this reason, Sjögren’s syndrome patients prefer to drink water instead of using other dry-mouth interventions. Water is widely accessible at low costs. Drinking water can temporarily relieve the subjective sensation of dry mouth [30, 31]. However, the effectiveness and longevity of this strategy are limited [27], because the viscosity of water does not change with increasing shear [32]. In contrast, the viscosity of saliva decreases with increasing shear. In practice, this allows saliva to be easily spread on the oral surfaces as well as to be retained and not easily washed off oral surfaces [32]. For the reason, saliva has important lubricating properties in contrast to water. As a consequence, the effectivity of drinking water as a dry-mouth intervention is limited compared to saliva.

Although the RODI-scores for the upper and lower lip were lower than other regions such as the posterior palate, anterior and posterior tongue and the pharynx, patients frequently moisten their lips (see Table 1). Maintaining moist lips appears to be important for patients and can be helped by the administration of simple water-based gels and ensuring humidification [33]. Another study concluded that scheduled use of ice water oral swabs and lip moisturizer with menthol may lessen thirst intensity and dry mouth [34].

Sjögren’s syndrome is an autoimmune disease that predominantly affects women. The female to male ratio of Sjögren’s syndrome is 10:1 [35]. This means that vast majority of female respondents in the present study (89%) is a good representation of the gender distribution of Sjögren’s syndrome in the Dutch population.

A possible limitation of the present study could be that the recruitment of the participants may have introduced a certain bias into the study. It can be assumed that Sjögren’s syndrome patients who visited the annual meeting of the patient federation suffer significantly from their disease and want their stories and problems to be heard. The response rate of these participants was 52%, whereas a response rate of 70–80% is envisaged to be ideal to eliminate a potential nonresponse bias [36], though the current response rate is comparable with the response rates of a previous study using a questionnaire (56%) which investigated health problems, health information sought and attendance of general practice in elderly patients with approximately the same age as our study population (70 years vs 64 ± 10 years in the current study) [37]. If a reminder was sent to the participants, then it could positively have affected the response rate. Several studies have shown that sending a reminder increased the response rate [38, 39]. However, sending reminders was not possible in the current study due to the General Data Protection Regulation (GDPA) restrictions with regard to collect personal data such as name and address. Therefore, it is possible that the study population in the present study is not representative for the total Sjögren’s population, as part of the opinion of the silent part of the population may not be present.

Additionally, patients attending the annual meeting may be more interested in their oral health than other Sjögren’s syndrome patients. This may have introduced an additional bias in the questionnaire responses that may have led to an overestimation of their perceived oral dryness.

A limitation of the current study could be that some specific interventions were not included in the questionnaire. For example, the low number of patients that reported the use of Xylimelts could be related to the fact that this intervention was not included. Also, the frequency and efficiency of the dry-mouth interventions were not included in the questionnaire. E.g., it is possible that Sjögren’s syndrome patients drank water many times a day, while they moistened their lips only one or twice a day. The perceived effectiveness of the dry-mouth interventions should also be evaluated, for example by asking the patients to rate this on a Likert scale. As the effectiveness of dry-mouth interventions might be related to the degree to which the salivary glands are still sensitive to stimulation [31], it is important that prospective studies also asses the relation between salivary flow rates and use of dry-mouth interventions.

## Main conclusions

The present study shows that Sjögren’s syndrome patients used a wide range of interventions to relieve their oral dryness, especially “drinking water’’ was a frequently used intervention care. As for the association between dry-mouth interventions with oral dryness and patients’ discomfort, only intra-oral dryness was significantly associated with the use of dry-mouth interventions. “Drinking water’’, “rinsing of the mouth”, and “drinking small volumes” had significant associations with the RODI-scores of the posterior palate, and anterior and posterior tongue, respectively, while the “use of a mouth gel’’ had a significant association with the RODI-scores of the inside cheeks. These results indicate that dryness of the posterior palate and the anterior and posterior tongue will influence Sjögren’s syndrome patients to use general dry-mouth interventions, such as “drinking water’’, “rinsing of the mouth” and “drinking small volumes”. On the other hand, dryness of the inside cheeks will cause patients to use a mouth gel. It can be concluded that Sjögren’s syndrome patients are more likely to use mouth gels when their inside cheeks were experienced as most dry, while they drank water, rinsed their mouth or drank small volumes if the posterior palate, anterior and posterior tongue were considered as dry. This finding has provided a deeper insight into the association between the use of dry-mouth interventions and mouth dryness, as intra-oral dryness affects dry-mouth perception and thereby also the use of the various dry-mouth interventions. Altogether, the therapeutic choice of a dry-mouth intervention by Sjögren’s syndrome patients seems to some extent to be related to dryness at specific oral regions.
